# Breast cancer risk reduction - is it feasible to initiate a randomised controlled trial of a lifestyle intervention programme (ActWell) within a national breast screening programme?

**DOI:** 10.1186/s12966-014-0156-2

**Published:** 2014-12-17

**Authors:** Annie S Anderson, Maureen Macleod, Nanette Mutrie, Jacqueline Sugden, Hilary Dobson, Shaun Treweek, Ronan E O’Carroll, Alistair Thompson, Alison Kirk, Graham Brennan, Sally Wyke

**Affiliations:** Centre for Research into Cancer Prevention and Screening, Cancer Division, Medical Research Institute, Level 7, Ninewells Hospital & Medical School, Dundee, DD1 9SY UK; Physical Activity for Health Research Centre, Moray House School of Education, St Leonard’s Land, University of Edinburgh, Edinburgh, EH8 8AQ UK; West of Scotland Breast Screening Service, Stock Exchange Court, 77 Nelson Mandela Place, Glasgow, G1 2QT UK; Health Services Research Unit, University of Aberdeen, Health Sciences Building, Foresterhill, Aberdeen, AB25 2ZD UK; Division of Psychology, School of Natural Sciences, Stirling University, Stirling, FK9 4LA UK; Department of Surgery, Dundee Cancer Centre, Mailbox 4, Ninewells Hospital & Medical School, Dundee, DD1 9SY UK; Physical Activity for Health Research Group, School of Psychological Sciences and Health, University of Strathclyde, Glasgow, G1 1QE UK; Institute of Health and Wellbeing, College of Social Sciences, Room 227 27 Bute Gardens, University of Glasgow, Glasgow, G12 8RS UK

**Keywords:** Breast cancer, Physical activity, Body weight, Alcohol, Sedentary time

## Abstract

**Background:**

Breast cancer is the most commonly diagnosed cancer and the second cause of cancer deaths amongst women in the UK. The incidence of the disease is increasing and is highest in women from least deprived areas. It is estimated that around 42% of the disease in post-menopausal women could be prevented by increased physical activity and reductions in alcohol intake and body fatness. Breast cancer control endeavours focus on national screening programmes but these do not include communications or interventions for risk reduction.

This study aimed to assess the feasibility of delivery, indicative effects and acceptability of a lifestyle intervention programme initiated within the NHS Scottish Breast Screening Programme (NHSSBSP).

**Methods:**

A 1:1 randomised controlled trial (RCT) of the 3 month ActWell programme (focussing on body weight, physical activity and alcohol) versus usual care conducted in two NHSSBSP sites between June 2013 and January 2014. Feasibility assessments included recruitment, retention, and fidelity to protocol. Indicative outcomes were measured at baseline and 3 month follow-up (body weight, waist circumference, eating and alcohol habits and physical activity). At study end, a questionnaire assessed participant satisfaction and qualitative interviews elicited women’s, coaches, and radiographers’ experiences. Statistical analysis used Chi squared tests for comparisons in proportions and paired t tests for comparisons of means. Linear regression analyses were performed, adjusted for baseline values, with group allocation as a fixed effect.

**Results:**

A pre-set recruitment target of 80 women was achieved within 12 weeks and 65 (81%) participants (29 intervention, 36 control) completed 3 month assessments. Mean age was 58 ± 5.6 years, mean BMI was 29.2 ± 7.0 kg/m^2^ and many (44%) reported a family history of breast cancer.

The primary analysis (baseline body weight adjusted) showed a significant between group difference favouring the intervention group of 2.04 kg (95% CI −3.24 kg to −0.85 kg). Significant, favourable between group differences were also detected for BMI, waist circumference, physical activity and sitting time. Women rated the programme highly and 70% said they would recommend it to others.

**Conclusions:**

Recruitment, retention, indicative results and participant acceptability support the development of a definitive RCT to measure long term effects.

**Trial registration:**

The trial was registered with Current Controlled Trials (ISRCTN56223933).

**Electronic supplementary material:**

The online version of this article (doi:10.1186/s12966-014-0156-2) contains supplementary material, which is available to authorized users.

## Background

Breast cancer is the most common cancer in Scottish women, accounting for 29% of female cancer cases and incidence is increasing (+11.1% between 2002 and 2012) [1].

Current estimates suggest that around 38% of breast cancer in post-menopausal women in the UK could be prevented by decreases in physical inactivity, alcohol consumption and body fatness [2]. Women meeting the World Cancer Research Fund prevention guidelines [3] show a 60% reduction in breast cancer compared to women meeting none [4]. In addition, observational studies show that breast cancer risk is lowered with intentional weight loss [5]. The Women’s Health Initiative intervention demonstrated that a 1% difference in body weight between intervention and control groups was associated with a 9% difference in breast cancer incidence after 8 years, suggesting even modest reductions in weight are beneficial [6]. Data from audits of bariatric surgery show that large weight losses are associated with large reductions in incidence of female cancers [7]. At any BMI, weight gain in adult life is associated with greater risk of breast cancer [8] with recent data suggesting that increases in adiposity between age 25 and post menopausal age are associated with a 33% increase risk in the disease [9]. A gain of 2–10 kg after age 50 years is associated with a 30% increase in breast cancer risk [10].

In Scotland, there are few initiatives directed at motivating change in diet and physical activity in women aged over 50 years despite recent data from the Scottish Health Survey (2012) reporting that 70% of women aged 55 to 74 years have a BMI >25 kg/m^2^ [11]. Furthermore, 42% of women do not achieve the recommendation of 150 minutes of physical activity per week. In 2012, 35% of Scottish women exceeded the recommended maximum weekly alcohol drinking levels [11].

Around 75% of Scottish women aged 50 to 70 years have accepted invitations to attend the NHS breast screening programme (NHSSBSP) and over 175,000 women are seen annually [12]. Fisher *et al*. [13] have reported that most women attending screening clinics are interested in receiving lifestyle advice but this finding requires further exploration. The NHSSBSP provides a unique opportunity for delivering an intervention aimed at breast cancer risk reduction which is consistent with the concept of the NHS as a health promoting service [14].

In addition, the absence of guidance may produce a ‘health certificate effect’ meaning patients receiving negative results (e.g. no cancer) may feel there is no need to modify their lifestyle. Failure to advocate lifestyle change in a breast cancer screening setting could endorse poor health behaviours [15] and the promotion of lifestyle advice within screening settings has been recommended [16]. This issue may be particularly relevant for body weight, where the lack of guidance to visibly obese patients may signal lack of medical concern.

This study aims to assess the feasibility of delivering a lifestyle intervention initiated within the breast cancer screening setting, in order to inform the design of a definitive randomised controlled trial (RCT) for weight management in post-menopausal women and consider the findings for health promotion in this setting. Feasibility and indicative outcomes will be reported in this paper.

## Methods

### Feasibility study design

This was a two-arm, feasibility study for a randomised trial conducted in two NHSSBSP settings. Following baseline measurement and assessment of eligibility, women were randomised to the intervention group (starting ActWell immediately) or the control group (offered lifestyle coaching at study end). We also conducted qualitative interviews with intervention group women, lifestyle coaches and radiographers to elicit their experiences of the programme.

### Participants and recruitment

The study was carried out in NHS Tayside and NHS Greater Glasgow and Clyde. Basic screening by mammography can take place either at a static breast screening unit (often hospital based) or on a mobile breast screening unit (mobile van). Approximately 5% of women screened are invited to recall clinics for further investigation because their basic screening mammogram shows some abnormalities or because other signs or symptoms were noted when they attended for basic screening. Breast screening colleagues advised against recruitment in mobile units (small space with little room for additional discussion) and the two other screening settings were explored.

In routine screening the protocol for recruitment comprised a letter from the clinical director of the local NHSSBSP endorsing the study plus a brief information sheet given to screening participants by clinic reception staff. This was followed by verbal endorsement and an invitation to join the study by radiographers at the end of mammographic screening. Clinic staff passed on contact details of interested women to the research team. The radiographer endorsement was timed to take around 1.5 minutes and was developed in conjunction with NHSSBSP staff.

Women attending recall clinics were sent a recruitment pack by post after receiving negative results. They received an endorsement letter, a brief information sheet and pre-paid reply slip and were contacted by the researchers on receipt of a positive reply slip.

Interested women were contacted and telephone-screened by the research team and if eligible [exclusion criteria: BMI < 20 kg/m^2^, reported contra-indicators to physical activity (e.g. recent post operation) or reported contra-indication to weight management (e.g. currently following a recovery programme for weight gain)] were sent a full participant information sheet and an invitation to complete baseline measures. Written informed consent was obtained at the baseline visit prior to assessments.

### Sample size

This was a feasibility study to test practical aspects (implementation, acceptability and feasibility) of study design and to help inform the calculation of effect sizes required for subsequent definitive RCT. With this in mind, we estimated that 80 women could be recruited, randomised (40 intervention and 40 control) and followed for three months and that this would provide sufficient data on programme implementation acceptability and feasibility to be able to make a decision as to whether moving to a full-scale trial was appropriate. For example, the acceptability of the intervention would be measured with precision of +/−12% given n = 40. More global outcomes such as overall recruitment would have a precision +/− 9% given n = 80. Although the study was not formally designed to test hypotheses, with 40 in each group, binary measures such as meeting a target would nevertheless have power of 80% to detect differences of 25% or more.

### Measures

Assessments were undertaken on two occasions namely at baseline (prior to intervention allocation) and at follow-up (after the 3 month intervention period).

At baseline the following data was collected by a researcher (independent of intervention delivery)

1. Demographic and clinical characteristics -age, marital status, ethnicity, educational achievement, employment status, socio-economic status (SIMD [17]), smoking status, reproductive history and family history of breast cancer. Height was also measured (using a Seca Leicester portable stadiometer).

The following additional procedures were undertaken at baseline and at 3-month follow up by the same researcher (blind to the participant’s group allocation):

2. Weight measured with the participant wearing indoor clothing and no shoes, using a calibrated Seca 877 digital scale.
3. Body mass index (BMI) was calculated – weight (kg)/[height(m)]^2^.
4. Waist circumference measured with a Seca 201 measuring tape, with participants in the standing position and the tape positioned midway between the lateral lower rib margin and the iliac crest. If these landmarks could not be identified, the measurement was taken at the level of the umbilicus. Two measurements were taken post exhalation and the mean recorded.
5. Blood pressure was measured using a Microlife BP 3BTO digital blood pressure monitor after the participant had been seated for 5 minutes. Two readings, or three if noted to be elevated, ≥1 minute apart, were taken and the mean reported.

In addition, a researcher administered questionnaire was used to record the following measures:

Diet – using the Dietary Instrument for Nutrition Education (DINE), a food frequency questionnaire of 19 groups of foods which account for about 70% of the fat and fibre in the typical UK diet [18]. Foods are grouped by nutrient content and dietary use and a score proportional to the fat or fibre content of a standard portion size, weighted by frequency of consumption is calculated. A fibre score of less than 30 (‘low’) is equivalent to a fibre intake of 20 g/day or less, whilst over 40 (‘high’) is equivalent to an intake of more than 30 g/day. A fat score of less than 30 is equivalent to a fat intake of 83 g/day or less (<35% of total energy intake for an average woman). An unsaturated fat score of up to 5 considered ‘low’ , and 10 or more considered ‘high’.

Alcohol – using 7 day alcohol recall [19]. Units of alcohol consumed per week and number of alcohol free days per week were calculated.

Physical activity and sitting time – using the International Physical Activity Questionnaire (IPAQ) short form [20]. Participants were asked to record how many days over the preceding seven days they had performed walking (in bouts of at least 10 minutes), moderate-intensity activities and vigorous-intensity activities and for how long. MET-minutes/week were then calculated according to the standard protocol (Table 1). Sitting time was also noted.

**Table 1:** **Scoring of the international physical activity questionnaire (IPAQ) short form**

Walking MET-minutes/week	3.3 x walking minutes x walking days
Moderate MET-minutes/week	4.0 x moderate-intensity activity minutes x moderate days
Vigorous MET-minutes/week	8.0 x vigorous-intensity activity minutes x vigorous-intensity days
Total physical activity MET-minutes/week	sum of Walking + Moderate + Vigorous METminutes/week scores

Psycho-social measures - self efficacy [21] and perceived health risk [22]. These results will be reported elsewhere.

The study administrator was responsible for monitoring all costs. Consumable and travel costs had to be checked against budget allocation, approved by the Principal Investigator (ASA) and paid via a single budget code enabling fine tracking of monthly and total costs. Intervention delivery costs were accounted for by Lifestyle coach salaries which were claimed on an hourly basis, checked by ASA in terms of travel time and the delivery time recorded (start and finish time) for each intervention participant.

Fidelity of the intervention was rated by a research assistant independent to the study using recordings from coaching sessions. To select intervention participants for recording a face to face interview, random numbers were generated from 1–40 (http://www.random.org/). The same process was repeated to select which participants to record a telephone interview with, and a new random number (1–6) was generated to select which of their scheduled phone calls to record.

A random sample of nine face to face interviews were transcribed and for each component of the intervention, a list of topics to be covered was prepared and fidelity to the intervention protocol was scored using a pre-prepared marking grid, e.g. introduction (10 items), weight management (10 items), physical activity (20 items) and diet (17 items). A similar procedure was used to rate the fidelity of the follow-up telephone calls.

To investigate participant acceptability, we asked 1 in 4 women who agreed to being contacted again to take part in semi-structured interviews which investigated their experience of the programme, which elements they found most helpful and which unhelpful and how they were able to incorporate changes into their everyday life. We also conducted semi-structured interviews with both lifestyle coaches and 2 paired interviews with 4 radiographers about their experience. In addition, programme acceptability questionnaires (with stamped addressed envelopes) were given to all participants after follow-up visits were completed. The questionnaire comprised ten questions each with a 5 point scale ranging from ‘very unhelpful’ to ‘very helpful’. Intervention participants were also asked to rate the helpfulness of intervention components (13 items).

Staff acceptability was investigated by semi-structured interviews with lifestyle coaches and radiographers.

### Randomisation

A randomised group allocation list was generated using nQUERY Advisor: 1:1, stratified by site and using mixed block size. Following baseline measures, women were randomly allocated by the study administrator to the intervention or control group. The study administrator then informed the participant and lifestyle coach of the group allocation.

### Intervention programme

All participants received a leaflet on breast cancer prevention [23]. Intervention participants were scheduled for a one hour lifestyle coaching session (face-to-face) and up to six fortnightly follow up telephone consultations for three months from one of two lifestyle coaches. Coaches were recruited who had a background of counselling and experience of advising on physical activity and dietary change. They were then trained in house by the programme developers over a 4 day period on study background and rationale, principles of weight management, study blinding, motivational interviewing techniques protocol delivery and relevant note taking. They were provided with individual participant folders and a structured programme for delivery including relevant note taking exercises pertinent to individual participant circumstances and specific goal setting challenges. Test coaching sessions were undertaken prior to actual study delivery.

An intervention information pack and pedometer based walking programme was provided (Table 2). Control group participants were offered a lifestyle coaching session after their three month follow up assessment.

**Table 2:** **Delivery and content of lifestyle coach face to face visit**

**Contact**	**Face to face**
**Duration**	60 min
**Who Delivers**	Trained coach
**Professional support**	Provide telephone and email contact details Identify next 2 phone call appointments
**Social support**	“Bring a buddy” offered, friend/partner/family member can be invited
**Summary of behaviour change techniques taught**	• Goal setting (modest, achievable, everyday life)
	• Action plans (implementation intentions)
	• Coping planning
	• Self-monitoring and feedback
**Goal setting**	Goals will be set for:
	• Weight
	• Physical activity
	• Food and drink (including alcohol)
	Implementation intentions agreed (when, where and how)
**Action plans coping plans self-monitoring**	Introduce activity focus
	• Provide pedometer
	• Pedometer/walking plan and diary
	• Offer home based activity tools (e.g. DVDs)
	• Explanation of self-monitoring procedures
	• Remind about goals
	• Provide written resource (back up of verbal information)
**Education and background**	20 minutes including interactive task
	Importance of lifestyle change and breast cancer risk
	• Brief background to which lifestyle factors increase risk and why
	- Weight
	- Physical activity
	- Food and drink
	- Alcohol
	• Consultant endorsement
	• Discussion of positive experience of weight loss
	• *Personal identification of weight category (interactive)*
	(SET weight goal; avoidance of weight gain or modest weight loss)
**Education – physical activity**	20 minutes, including interactive walk and talk
	• Activity and inactivity
	• *Demonstration of brisk walking + pedometer (interactive)*
	• Personalised Walking plan (to fit with everyday life alternative if participant wishes)
	• Physical activity guidelines
	• Local facilities
	(SET physical activity goal)
**Education - diet**	20 minutes including interactive task
	• Weight gain and the risk of breast cancer
	• Energy dense food and snacks
	• Breakfast
	• Sugary and alcoholic drinks
	• *Alcohol – measurement task (interactive)*
	(Personalised plans and SET food and drinks goal)
Telephone intervention for fortnightly calls	
**Contact**	Telephone, fortnightly
**Time line**	Following on from face to face contact until 3 month follow up assessment (6 calls total)
**Duration**	15 min
**Who delivers**	Trained coach
**Professional support**	Make appointment for next telephone call but re-iterate you can respond to questions before this call as they arise
**Content**	General exchange about mental and physical health Elicit participant’s overview on progress and changes made Re-enforce importance of modest behaviour change for health benefit Importance of change and building towards 3 month weight target Importance of remaining active Re-enforce guidance for breakfast, energy dense foods and snacks, sugary drinks, alcohol
**Motivational approaches**	Check self-monitoring records Identify perceived diet/activity challenges Identify self-assessed motivations, confidence, ambivalence and personal value re: diet and physical activity
**Personal goals (implementation intentions)**	Continue to focus on short term implementation intentions and review these at next call
**Setting long term goals**	Identify perceived achievements and summarise success Re-evaluate confidence, motivation and importance of changes made

The intervention aimed to help women increase physical activity, modify their diet, lower their alcohol intake, and set individual weight management goals (weight loss or avoidance of weight gain). The sessions took place in the research institutions (not screening clinics).

The face to face session was designed to be interactive and included a 10 minute walk and talk session (measured university corridors), self-identification of BMI from standard charts and measurement of one standard unit of alcohol using a wine coloured liquid. Intervention participants were invited to bring a support person to the meeting.

Women received personalised help to learn the impact of lifestyle behaviours on breast cancer risk and how to make any necessary changes in their lives, set personal goals, tips on how to make changes habitual by talking through their personal routines, and relapse strategies for times of deviation.

Motivational interviewing techniques explored self-assessed confidence to change and self-perceived benefits. Behavioural techniques, known to be effective in changing physical activity and diet, were used by the lifestyle coaches [24,25]. These focussed on goal setting, action and coping plans and implementation intentions [26]. The lifestyle coach assisted in goal setting to ensure goals were challenging and achievable. Goals were set for three domains of action (diet, physical activity and weight) through discussion with participants. All goals were developed from current behaviours and modest and practical changes identified according to personal lifestyles.

The importance of recording and self-monitoring pedometer data, diet and drink logs and weekly body weight was emphasised. These parameters also formed the basis for the intervention phone calls which aimed to be 15 minutes in duration and checked wellbeing, progress on implementation intentions, self-monitoring behaviours and reviewed individual actions. The phone call appointment was scheduled at each meeting with emphasis on recording and reporting, but coaches indicated that flexibility was possible given work and vacation schedules.

### Statistical analysis

Quantitative data analysis was undertaken using SPSS (Version 21.0, IBM Corporation, Armonk, NY, USA). Descriptive statistics allowed characterisation of the cohort. For indicative outcomes, Chi squared tests were used for comparisons in proportions and paired t tests for comparisons of means. Linear regression analyses were performed, adjusted for baseline values, with group allocation as a fixed effect. In the case of missing values, a Baseline Observation Carried Forward Analysis was undertaken for the primary outcome (weight change).

### Qualitative analysis

The interview data were audio-recorded with participant consent and transcribed *verbatim*. NVivo software (Version 9, QSR International, Melbourne, Australia) was used to assist data coding and organisation. Transcripts were analysed thematically using the Framework Approach, a case and theme based approach that allows themes to be presented in a matrix with cases [27]. For participants, the themes coded were based on their experiences of the programme in relation to recruitment, why they took part, and what aspects of the programme they felt worked for them. For coaches and radiographers the themes were based on the extent to which they felt able to deliver the programme as intended.

The study received ethical approval from the East of Scotland Research Ethics Service REC reference no. 12/ES/0087.

## Results

### Recruitment and retention

**Figure 1:**
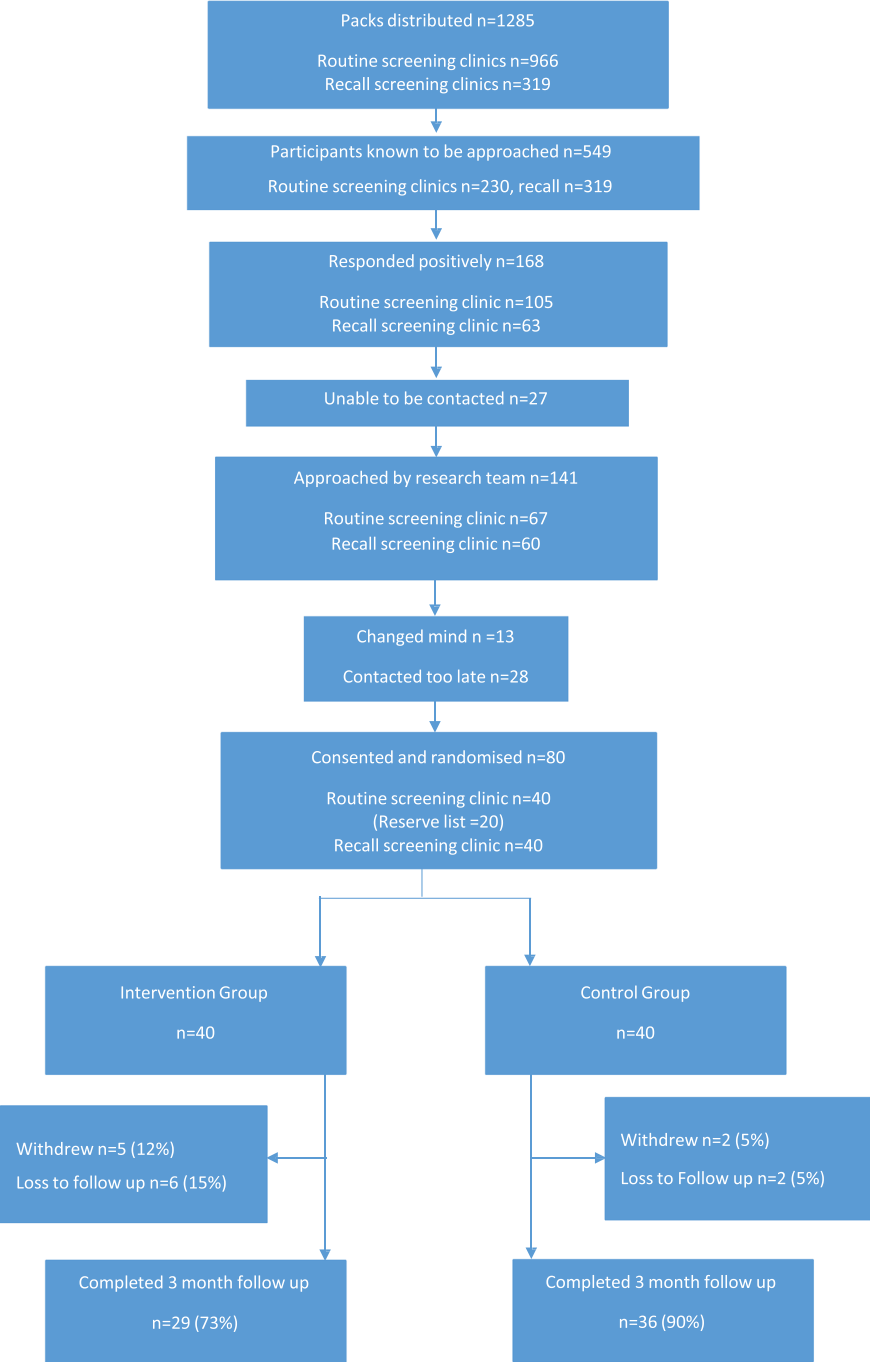
**ActWell CONSORT flowchart.**

In the routine screening clinics 966 recruitment packs were handed out by reception staff over a 7 week period (Figure 1). Written records kept by radiography staff showed that 230 women had received the study endorsement. Of these, 100 (43%) expressed positive interest, 46 (20%) expressed possible interest and 84 (37%) were not interested. Attempts were made to contact 105 women; including 5 women not on the radiographer lists who may have expressed interest through recruitment packs alone or may have spoken with radiographers but their interest was not recorded at the time. Of the 105 women, 25 (24%) could not be contacted, 7 (6%) changed their mind, 13 (12%) were contacted as recruitment was ending and 60 (56%) were ready to participate (20 were then put on a reserve list following the achievement of our pre-set target of 40).

Recruitment packs were sent to 319 attendees of recall clinics over a 12 week period and 63 (20%) women returned the pre-paid reply slip to the study team indicating a willingness to participate. The study team then contacted those women who had expressed a willingness to participate, however, 2 (3.2%) could not be re-contacted, 6 (9.5%) changed their minds and 15 (23.8%) responded positively after the recruitment target (40) had been reached. No women were excluded (i.e. none met the exclusion criteria).

At 3 months, 65 study participants undertook follow up assessment (81.3% retention). No significant difference was detected in % follow up by group, (73% intervention versus 90% control). In the intervention group, 3 women dropped out prior to the first intervention session, 2 during the telephone contact period and 6 (15%) who had completed the intervention programme were lost to follow up. Reasons given for withdrawal were ill health by 4 participants (intervention group) and one participant (control group) said she withdrew due to group allocation. More women from routine screening clinics (n = 35, 88%) completed the study than from recall clinics (n = 30, 75%).

The mean age of participants was 58 ± 5.6 years (Table 3) and 44% reported a family history of breast cancer. There were no significant differences in ethnicity, marital status, deprivation category or employment status between the groups. The control group was more educated than the intervention group (p = 0.02). The mean BMI was 29.2 ± 7.0 kg/m^2^ and 71% had a BMI >25 kg/m^2^ (Table 4).

**Table 3:** **Socio-demographic characteristics at randomisation**

	**Intervention (n = 40)**	**Control (n = 40)**	**All (n = 80)**
**Age (years) mean (SD)**	58.4 (6.0)	58.1 (5.5)	58.3 (5.78)
Range	50 – 75	50 – 69	50 – 75
**Marital status**			
Single	4 (10.0%)	3 (7.5%)	7 (8.8%)
Married/co-habiting	29 (72.5%)	24 (60.0%)	53 (66.3%)
Divorced/widowed/separated	7 (17.5%)	13 (32.5%)	20 (25.0%)
**Ethnicity**			
White	39 (100.0%)	39 (97.5%)	78 (98.7%)
Asian/Asian British	0 (0.0%)	1 (2.5%)	1 (1.3%)
Highest educational qualification*			
Secondary school	18 (45.0%)	11(27.5%)	29 (36.3%)
Other professional/technical qualification after school	18 (45.0%)	13 (32.5%)	31 (38.8%)
University/post-graduate degree	4 (10.0%)	16 (40.0%)	20 (25.1%)
**Employment status**			
Retired	18 (45.0%)	14 (35.0%)	32 (40.0%)
Employed full-time	9 (22.5%)	17 (42.5%)	26 (32.5%)
Employed part-time	8 (20.0%)	6 (15.0%)	14 (17.5%)
Unemployed	5 (12.5%)	2 (5.0%)	7 (8.8%)
Student	0 (0.0%)	1 (2.5%)	1 (1.3%)
**SIMD# (quintiles)**			
1 (most deprived)	7 (17.5%)	3 (7.5%)	10 (12.5%)
2	9 (22.5%)	5 (12.5%)	14 (17.5%)
3	8 (20.0%)	9 (22.5%)	17 (21.3%)
4	6 (15.0%)	9 (22.5%)	15 (18.8%)
5 (least deprived)	10 (25.0%)	14 (35.0%)	24 (30.0%)

^#^Scottish Index of Multiple Deprivation.

*p < 0.05, significant.

**Table 4:** **Reproductive, medical and clinical characteristics at randomisation**

	**Intervention (n = 40)**	**Control (n = 40)**	**All (n = 80)**
**Body weight (kg) mean (SD)**	78.0 (16.5)	73.6 (18.7)	75.8 (17.7)
**BMI (kg/m** ^**2**^ **) mean (SD)**	30.2 (6.5)	28.2 (7.4)	29.2 (7.0)
**Smoking status**			
Current	4 (10.0%)	4 (10.0%)	8 (10.0%)
Never smoked	18 (45.0%)	25 (62.5%)	43 (53.8%)
Ex-smoker	18 (45.0%)	11 (27.5%)	29 (36.3%)
**Cigarettes per day**			
**Mean (SD) (consumers only)**	11.3 (4.4)	9.5 (7.9)	10.4 (6.0)
**Number of children**			
0	8 (20.0%)	8 (20.0%)	16 (20.0%)
1**–**2	21 (52.5%)	24 (60.0%)	45 (56.3%)
3-4	11 (27.5%)	8 (20.0%)	19 (23.8%)
**Number of pregnancies**			
0	6 (15.0%)	7 (17.5%)	13 (16.3%)
1**–**2	16 (40.0%)	20 (40.0%)	36 (45.1%)
>3	18 (45.0%)	13 (32.5%)	31 (38.9%)
**History of breastfeeding**	18 (45.0%)	26 (65.0%)	44 (55.0%)
**Family history of breast cancer**	14 (35.0%)	21 (52.5%)	35 (43.8%)

Baseline characteristics of participants who completed the study compared to those who did not complete showed significant differences by employment status (p = 0.006). Completers were more likely to be retired and not registered as unemployed (although there was no difference in age between groups). There were no differences in baseline characteristics between participants recruited from recall or routine clinics.

### Intervention delivery

In the intervention group, 37 women underwent the face-to-face session (3 withdrew prior to being seen). One participant took part with a buddy. Thirty-two participants completed all 6 fortnightly calls (78%), including 6 who were subsequently lost to follow up. Five participants received fewer calls, including one who requested no calls and two who dropped out after the first few calls were received. The mean duration of the face to face consultation was 90 minutes (range 65 to 130 minutes); the planned protocol time was 60 minutes. Mean duration of telephone consultation was 22 minutes (range 10 to 54 minutes); the planned protocol time was 15 minutes. The lifestyle coaching session offered to the control group after follow up measures was delivered to 30 participants (83%).

The fidelity assessments showed that the intervention delivery appeared close to protocol (scoring 68 to 78% for the domains of introduction, weight management, physical activity, and diet). The fidelity of the telephone sessions were also rated close to protocol. Deviations from the protocol included the coach, not participant, setting goals, not discussing the intervention in terms of personal wellbeing and limited discussion of coping planning.

The intervention costs were £17 265 comprising £5 200 for training, plus intervention costs of £301 per participant.

### Indicative outcomes

The study was not fully-powered but indicative intervention effect data are presented below.

### Physical measures

Mean weight loss at 3 months in the intervention group was 2.04 kg (95% confidence interval (CI) -2.98 kg to −1.11 kg) and in the control group was 0.04 kg (95% CI −0.82 kg to 0.75 kg). The primary analysis (with adjustment for baseline body weight) showed a group difference in weight loss of 2.04 kg (95% CI −3.24 kg to −0.85 kg) in favour of the intervention group. These differences remained significant when a baseline observation carried forward assessment was taken to allow for missing cases. Greater reductions in BMI and waist circumference were also detected in the intervention group than in the control group at three months (Table 5).

**Table 5:** **Changes in anthropometric measures and blood pressure from baseline to 3 months by randomisation group**

		**Intervention**			**Control**			
				**Difference to baseline**			**Difference to baseline**	**Between group differences** ^**#**^
		**n**	**Mean (SD)**	**Mean (95% CI)**	**n**	**Mean (SD)**	**Mean (95% CI)**	**Mean (95% CI) p value**
**Body weight (kg)**	Baseline	40	78.0 (16.5)		40	73.6 (18.7)		−2.04(−3.24 to −0.85)*
	3 months	29	73.0 (14.3)	−2.04(−2.98 to −1.11)*	36	72.8 (19.3)	−0.04(−0.82 to 0.75)	p = 0.001
**BMI (kg/m** ^**2**^ **)**	Baseline	40	30.2 (6.5)		40	28.2 (7.4)		−0.77(−1.24 to −0.31)*
	3 months	29	28.1 (5.6)	−0.79(−1.16 to −0.41)*	36	27.9 (7.5)	−0.03(−0.33 to 0.28)	p = 0.002
**Waist Circumference**	Baseline	40	98.7 (14.9)		40	93.6 (15.4)		−3.53(−5.50 to −1.57)*
**(cm)**	3 months	28	91.4 (11.6)	−4.41(−6.06 to −2.75)*	36	92.0 (15.6)	−0.79(−1.98 to 0.40)	p = 0.001
**Mean systolic blood**	Baseline	40	129.4 (13.1)		39	129.4 (17.7)		−2.81(−9.27 to 3.65)
**pressure (mmHg)**	3 months	28	130.9 (12.1)	0.59(−3.58 to 4.76)	34	132.6 (19.0)	4.05(−1.52 to 9.61)	p = 0.388
**Mean diastolic blood**	Baseline	40	78.6 (9.5)		39	78.8 (9.5)		−0.77(−4.22 to 2.69)
**pressure (mmHg)**	3 months	28	79.4 (6.1)	1.10(−1.46 to 3.66)	34	80.0 (9.9)	2.01(−1.06 to 5.07)	p = 0.659

^#^Adjusted for baseline value *significant, p < 0.05.

### Reported behaviours

The intervention group reported *more* activity and *less* sitting time than the control group (Table 6). No between group differences were detected in dietary intake.

**Table 6:** **Changes in self-reported dietary intake and physical activity (PA) from baseline to 3 months by randomisation group**

		**Intervention**			**Control**			**Between group differences** ^**#**^
		**n**	**Mean(SD)**	**Difference to baseline mean (95% CI)**	**n**	**Mean(SD)**	**Difference to baseline mean (95% CI)**	**Mean (95% CI) p value**
**Continuous PA score per week (MET-min per week)**	Baseline	39	1573(1651)		39	1810(1716)		
	3 months	29	2437(2157)	1021(256 to 1787)*	36	1469(1600)	−249(−849 to 351)	1111(233 to 1990)* p = 0.014
**Sitting hours per week**	Baseline	37	43(19)		39	39(16)		
	3 months	29	34(14)	−6.85(−12.98 to −0.71)*	36	45(25)	5.23(−2.92 to 13.38)	−11.48(−21.37 to −1.59)* p = 0.024
**Fat consumption score**	Baseline	40	28.4(13.9)		40	25.8(10.5)		
	3 months	29	24.4(7.9)	−4.38(−7.99 to −0.77)*	36	22.4(7.5)	−2.33(−4.98 to 0.32)	0.20(−2.76 to 3.16) p = 0.894
**Unsaturated fat score**	Baseline	40	8.0(2.2)		40	8.4(2.1)		
	3 months	29	8.7(1.6)	0.83(−0.01 to 1.67)	36	8.5(1.8)	0.06(−0.66 to 0.77)	0.35(−0.46 to 1.16) p = 0.390
**Fibre food consumption score**	Baseline	40	31.7(12.9)		40	28.9(8.2)		
	3 months	29	33.3(9.2)	1.21(−1.35 to 3.77)	36	28.2(9.4)	−0.25(−2.44 to 1.94)	2.40(−0.74 to 5.54) p = 0.131
**Increased fruit intake n(%)**	3 months	29	7(24.1)		36	5(13.9)		p = 0.521
**Increased vegetable intake n(%)**	3 months	29	16(55.2)		36	12(33.3)		p = 0.194

^#^Adjusted for baseline value *significant p < 0.05.

Most (61%) reported regularly consuming alcohol, although intakes ranged widely (0 to 66 units per week). The number of alcohol free days increased in both groups and although consumption reduced in both groups this only reached significance in the control group which included one drop out with high intakes at baseline (Table 7).

**Table 7:** **Changes in alcohol consumption only from baseline to 3 months by randomisation group**

		**Intervention**			**Control**			**Between group differences** ^**#**^
				**Difference to baseline**			**Difference to baseline**	**Mean (95% CI)**
		**n**	**Mean(SD)**	**Mean (95% CI)**	**n**	**Mean(SD)**	**Mean (95% CI)**	**p value**
**Units alcohol per week**	**Baseline**	22	10.4 (13.7)		27	10.8 (6.2)		
	**3 months**	18	5.3 (5.1)	−2.29 (−4.74 to 0.16)	26	5.6 (5.9)	−5.1 (−7.58 to −2.63)*	1.20(−1.81 to 4.21)
								p = 0.424
**Alcohol free days per week**	**Baseline**	22	4.8 (2.3)		27	3.8 (1.2)		
	**3 months**	18	5.4 (1.1)	1.1 (0.28 to 2.0)*	26	5.2 (1.9)	1.3 (0.56 to 2.06)*	0.05(−0.84 to 0.94)
								p = 0.910

^#^Adjusted for baseline value.

*significant p < 0.05.

### Programme acceptability

Of seventeen women invited to participate in semi-structured interviews, 14 completed interviews.

The recruitment process and the programme were highly acceptable to women who took part in interviews. Examples of views on acceptability of the recruitment process and delivery of the programme are given below.

#### Views on recruitment process

“I think doing it that way you feel it's your decision, it's not as if somebody's selling you something, you know, trying to force you into it. I mean, I picked up the leaflet and while I was waiting to be seen by the nurse and when I went in I went I really like the look of this and she says well I’ll put your name forward for it”. (DP1, Age 53)

#### Reasons for taking part

*“Well I went down for a mammogram and there was leaflets there inviting you to participate so I said I would do it because my son is a research doctor and I know that you need people too, for research… Well I was happy to take part in research and also I wanted to see how it would benefit my health you know”.* (DP2, age 69)

#### Information about link between lifestyle factors and cancer risk was new

"*There was nothing new to me no not really because I know what I should do. [But the cancer risk information] I was quite interested with that yeah. I hadn’t actually got that side of it that you know putting weight on round [your waist] and all the rest of it. I mean, I knew that that's bad for your heart and so on, I hadn’t really thought about it in connection with the breast cancer".* (DP6, age 61)

#### Coaches’ non-judgemental positive approach was appreciated

*“To actually see the life coach and then be in touch with somebody and somebody saying to me right ok what have you done, that I do need and that side of it was actually quite positive”. (DP6, age 61)*

#### Appreciation of practical advice on small, sustainable, changes that could be made in the context of everyday life

*“I had got used to [the step goals] quite easily… 3 days 1500 [steps] for a week you know. And you did that for 2 weeks it went up very slowly and it was quite manageable”. (PG6, age 57)*

*“if I don’t make my lunch at 2 o’clock I’ll be going right well if I eat [later] then I'm not going to be hungry at dinner time so I might be as well having a snack now at 2 so I’ll have my dinner, I structure it that way so it's not in my face but it's there, it's a whole attitude change”. (PG2, age 54)*

#### Telephone contacts highly valued, in particular their non-judgemental approach

*“The mad thing is I mean at my age you kind of wanted to please [the lifestyle coach], I've done it, I've done it, I'm not a failure. I kept my targets going for you and yeah I found she was very good and as I say if there was a problem like I just wasn’t happy with the sit ups she gave me alternatives”. (PG1, age 61)*

*“You didn’t feel as if she was disapproving or, judgemental, is probably the best word. She wasn’t judgemental about it at all, so no I thought she was very nice”. (PG1, age 61)*

#### Would have liked a longer programme or less abrupt completion

*“It would be good if there was maybe another follow up a bit later on instead of just it being finished and that's it. It would be good if maybe somebody was checking up in 6 months’ time”. (DP1)*

Women acknowledged the usefulness of the leaflet, but emphasised the importance of endorsement of the programme by staff. They identified three main reasons for taking part: to help out with research; because they had a family member or friend affected by cancer; the opportunity to improve their health. It was also notable that, for some, the relationship between lifestyle factors in relation to breast cancer (rather than heart disease) was being raised for the first time.

Women enjoyed the programme and the approach taken by lifestyle coaches. The phone calls were also viewed positively. The pedometer was highly valued and described as a useful way to learn how to change behaviour. Telephone support was thought to be very useful but some women felt the programme should have been longer.

Anonymised responses from the programme acceptability questionnaires were returned by 57 participants (88% of completers). The majority (73.4%) said they were very satisfied with the programme and 70% said they would recommend it to others. In terms of “helpfulness” of the intervention components, the pedometer was rated “very helpful” by 84%, followed by face to face contact and telephone support (both 79%) (Table 8).

**Table 8:** **Acceptability of intervention measures**

	**Intervention n = 25(%)**
Remembered receiving any leaflets or booklets from the ActWell lifestyle coach	23(92.0)
Set personal goals to assist with physical activity levels	24(96.0)
Discussions/questions about your confidence in changing your lifestyle very/quite helpful	21(87.5)
Set personal goals to assist with weight management	23(92.0)
Found the pedometer very helpful	21(84.0)
Face to face contact with the lifestyle coach very helpful	19(79.2)
Telephone contact with the lifestyle coach very helpful	19(79.2)
Set personal goals to assist with diet	19(76.0)
Found the leaflets/booklets provided by the lifestyle coach very helpful	17(73.9)
Found the personal goals (physical activity) very useful	16(66.7)
Found the personal goals (diet) very useful	12(63.2)
Found the personal goals (weight management) very useful	14(60.9)
Set personal goals to assist with alcohol use	5(20.8)
Found the personal goals (alcohol) very useful	4(80.0)

Both lifestyle coaches completed interviews. Both highlighted the usefulness of the information pack and the benefits of doing face-to-face meetings prior to telephone contact. They also mentioned difficulties arranging the counselling phone calls and the time this took.

Two paired interviews with radiography staff in Dundee and Glasgow were undertaken. Radiographers highlighted that they did not feel equipped to explain the programme to women and some did not feel it appropriate for them to take on the role of programme endorsement or give assistance with recruitment. The radiographers’ reported fidelity to their prescribed role was low. They said time was the biggest problem, resulting in staff feeling under pressure and incapable of elaborating on the ActWell programme or answering questions.

## Discussion

This study has demonstrated the feasibility of recruiting and retaining women in a lifestyle intervention trial initiated through the breast screening setting with indications of a favourable effect on body weight and related outcomes at 12 weeks and participant acceptability.

We acknowledge that the current study is not a fully powered trial but results are encouraging and warrant further investigation in a fully powered trial of longer duration. Our recent fully powered weight loss trial initiated in a colorectal cancer screening setting achieved similar weight loss (2.10 kg (CI −2.57 kg – 1.63 kg) at 12 weeks, but with further monthly support for 9 months achieved weight loss of 3.50 kg (SD 4.91) at one year, highlighting the importance of offering longer intervention support [28]. It is notable that in the current study the women interviewed had a very positive experience of the ActWell programme.

In addition, it is important to remember that that lifestyle interventions in the screening setting will only reach people who choose to participate in such programmes. In many cases, this means people from areas of low social deprivation and with better access to affordable health care. However, the study participants included those from a wide range of social backgrounds. It is notable that almost half reported a family history of breast cancer and this may be a cue for interest in prevention opportunities.

One of the strengths of the study is utilising an existing and relevant recruitment setting but it is clear that further work is needed to optimise this opportunity. Within the two screening settings explored it is notable that the best recruitment was attained in routine clinics when women had received the recruitment pack AND the study was endorsed by the radiographer. However, it was clear that the radiographers did not feel comfortable with giving endorsement and asking about women’s interest in the programme (demonstrated by qualitative interviews and the finding that only 230 of 966 women were recorded as being asked about the study). It is recognised that there are challenges in discussing interventions which include weight management. Staff may be uneasy about confronting patients with the issue of body size, especially if their training has not included guidance on how to broach the topic or effective intervention techniques, or if staff have personal weight problems [29].

The benefits of wide engagement and excellent response when radiographers were involved in endorsement and recruitment mean that routes to minimize study burden by radiographers are desirable (and may be aided involving the wider screening team staff). Our recruitment at recall clinics using a mailed approach (due to high anxiety exhibited during the recall clinics visit) was lower than routine clinics although better than that reported by Evans *et al*. [30] of 12.5% (40/340) for high risk women entering a lifestyle study, and 6.3% entered a randomised study of two weight loss programmes. The reasons for the lower recruitment than the current response are unclear but may relate to the content of the ActWELL programme which clearly embraced positive aspects of physical activity, diet and behavioural support offering a much wider lifestyle approach than highlighting weight loss per se.

Despite reporting a family history of breast cancer, several commented in the follow up interviews that they had been unaware of the association between excess body weight, physical activity and modest alcohol intake with breast cancer risk. This highlights a gap in women’s awareness of breast cancer risk reduction and serves as a reminder of the under developed potential of screening clinics for health promotion opportunities.

A recent review of critical research gaps and translational priorities for the successful prevention and treatment of breast cancer has highlighted the need for more research on how to implement sustainable lifestyle changes in women [31]. While there may be many "teachable moments" that could be used to assess and initiate changes in physical inactivity, body weight and alcohol by health professionals (to complement public health campaigns), there is little evidence that lifestyle is routinely discussed within breast cancer screening settings [32] (although these have been delivered in the colorectal cancer screening setting [32,33]). Effective interventions delivered in this setting also offer an important opportunity to contribute to the reduction of the overall burden of lifestyle related diseases. To our knowledge this is the first lifestyle intervention delivered and evaluated within a national breast screening programme.

In terms of implications for policy and practice our overall study retention was reasonable and outcomes were good at three months but further research is needed to consider how to improve retention and sustain change beyond this time frame. Our previous research [28] on lifestyle in a screening setting showed a loss to follow up of 4% at 12 weeks (similar to the control group in the current study), followed by total loss of 7% at 12 months. We believe the self-monitoring and feedback from counsellors through minimal but regular contact (telephone calls) over a 12 month period supported retention. In terms of real life implementation, two year routine breast screening appointments could provide a potential opportunity to help women monitor weight with feedback from within a supportive healthcare setting for sustaining long term behaviour change.

Why there was higher loss to follow-up in the intervention group of the current study is unclear. We did not have ethical approval to write to women who had dropped out and although we did have approval to telephone them, we were unable to contact them. We do not, therefore, have their reasons for dropping out. It may that some women tried the intervention, found it wasn’t for them and then ceased contact with the study, a situation that clearly doesn’t affect the control group. This was seen for men in the Football Fans in Training (FFIT) lifestyle change trial [34] where retention at 12-weeks was 88% in the intervention group and 93% in the control group, with men in the intervention group generally dropping out because they decided the intervention was not for them. The difference in retention between groups in the FFIT study was modest but was 17% in the current study, though the difference didn’t reach statistical significance. In a small feasibility study such as the current one, a few extra participants staying in or dropping out can make relatively large difference to the percentages (FFIT involved 747 men; this study 80 women) so we need to be careful in how we interpret the absolute difference in retention between groups. However, for the current study our conclusion must be that differential loss to follow-up is a potential risk for a future trial, that this may be due to the nature of the intervention and that any future trial will need to take steps to avoid loss to follow-up in both groups but especially the intervention group.

In addition, it is important to consider how a successful intervention might be delivered in the non–trial setting given the paucity of NHS staff trained in lifestyle interventions and issues of cost effectiveness. These are similar challenges to those of smoking cessation which have been addressed in many clinical settings by ready access to a NHS smoking cessation counsellor (in addition to offering community group support etc.). This approach also deserves consideration with respect to weight management.

## Conclusions

It is feasible to deliver a minimal contact lifestyle programme for women in conjunction with the NHSSBSP with positive indicative effects on body weight, physical inactivity, alcohol intake and participant acceptably measures. The results warrant further exploration by a definitive randomised controlled trial.

## Abbreviations

BMI: Body mass index
NHS: National health service
NHSSBSP: NHS breast screening programme
RCT: Randomised controlled trial
SIMD: Scottish index of multiple deprivations
DINE: Dietary instrument for nutrition education
IPAQ: International physical activity questionnaire
SPSS: Statistical package for social scientists
CI: Confidence interval
SD: Standard deviation
